# Simulation-based perioperative anaesthesia information management practice: cross-sectional study

**DOI:** 10.1097/MS9.0000000000000471

**Published:** 2023-04-07

**Authors:** Belete Muluadam Admassie, Yonas Admasu Ferede, Zemenay Ayinie Mekonnen, Menarguachew Atanaw Sisay, Misganaw Degu Worku

**Affiliations:** Departments ofa Anesthesia; b Surgery, College of Medicine and Health Sciences, University of Gondar, Ethiopia

**Keywords:** anaesthesia documentation, information management, record

## Abstract

Perioperative anaesthesia record completeness is very essential skill of the anaesthesia profession at the time of operation in the health setting. During perioperative, anaesthesia care sometimes there may be missing important information of the patient, medication taken or planned. This study aimed to improve perioperative anaesthesia information management practice.

**Methodology:**

Pre-interventional and post-interventional cross-sectional study was conducted from 21 June to 25 July 2022 on 164 anaesthesia record filled by 51 anaesthesia care provider in pre-interventional and post-interventional phase. Data were collected using a semi-structured questionnaire and the data entered by Epi-data software (version 4.6) and analyzed by using SPSS version 26. For all indicators, the projected completion rate was 100%. Indicators with completion rates of greater than 90% were classified as acceptable, while those with completion rates of 50% were seen as urgently needing improvement.

**Results:**

Pre-interventional result: among all indicators, none of the indicators had 100%, completeness rate. Postoperative nausea and vomiting management orders, the names of the surgeon and anaesthetist, the location of the intravenous cannula, the maintenance of anaesthesia, the total amount of fluid supplied, the content of the consent discussion, and null per ose status, age, and weight of the patient were some of the markers that were identified below average (50%) and in need of significant improvement. Post-interventional result: when compared with the pre-interventional result, their documentation skills were improving after discussions with stakeholders and the relevant bodies; however, none of the indicators attained 100% completion rate.

**Conclusion and recommendation:**

Even after the interventions, the desired completion rate was not attained. As a result, it requires ongoing instruction on perioperative anaesthesia information management according to the standard perspectives.

## Introduction

Highlights

- Anaesthesia documentation is an integral part of a patient’s journey.
- Perioperative anaesthesia information management were below standards.
- After intervention most of the indicators were improved.

The main goal of anaesthesia documentation is to record precise and thorough information in order to convey a patient’s anaesthetic experience. In addition, references made for medico-legal purposes because anaesthetists are more likely to encounter various medico-legal problems may result from inadequate recording and communication with other staff members, patients, and family members. The quality of care given to patients might suffer from inadequate documentation, which is also crucial for quality improvement, research, and external organization review. Documentation also plays a crucial part in medico-legal elements. Workload, the use of tools or manual recording, the interest of the practitioner, the use of tools, the accessibility of information, and so forth are some of the elements that influence the standard and practice of documentation^1^,^2^.

In accordance with the World Federation of Societies of Anesthesiologists’ (WFSA) and the Association of Anaesthetists of Great Britain and Ireland’s (AAGBI) criteria, the degree of manual anaesthetic record completeness was found to be substandard in several studies^2^.

The anaesthesia information record forms an integral part of a patient’s journey through the hospital system^3^. It is a clinical, scientific, legal, and administrative record pertaining to patient care that presents pertinent data in a sequential manner, justifies the diagnosis, carries out the recommended course of action, and achieves the desired outcome^4^,^5^.

A study done in Toronto on anaesthetic record completeness and accuracy among 124 participants stated that charting of data to the anaesthetic record remained incomplete and inaccurate in all groups based on level of training, age, and number of years in practice^6^. A similar study done in South Africa on 284 records regarding anaesthetic record completeness showed that less than one third of all records were complete and legible, and for one quarter of all anaesthetics, no record of any kind was made^7^.

A comparative study done in Nigeria on the effect of teaching on the completeness of the anaesthesia record charts for obstetric subarachnoid blocks showed that from a total of 500 anaesthetic record, the average percentage completion of anaesthetic charts before the lecture (pre-intervention); first, second, third, and fourth audits (post-intervention) were 56.1%, 70.1%, 78.1%, 81.3%, and 87.7%, respectively^8^.

Clinical audit done in Ethiopia on anaesthetic record completeness at a university teaching hospital operation theatre in a low-resource setting on 120 anaesthetic records stated that the level of record completeness was less than 50% for the content of consent, pre-induction vital signs, intraoperative vital signs, start and end of both anaesthesia and surgery, type of regional anaesthesia, intraoperative blood loss, and order for postoperative management^9^.

Therefore, the demands of perioperative anaesthesia documentation are growing well; however, there is less adequate documentation form and there is interpersonal variation during documentation. So, this study may help to enable anaesthetists to practicing the best evidence- based perioperative anaesthesia information management techniques, to guide the practitioner to meet a given standard of care, to remind clinicians of the basic minimum requirements for safety and quality of care for patients, and to identify the gaps in current practice of anaesthesia information documentation compared with the standards.

## Methodology

The institutional ethical review committee granted its approval after receiving ethical clearance. We received consent from clinical directors of the study setting to retrieve patient chart and we take verbal informed consent from study participants. The STROCSS reporting guidelines were followed^10^, and this work was registered with the UIN research registry.

### Study design, period and area

From 21 June to 25 July 2022, an pre-interventional and post-interventional cross-sectional study was carried out was conducted to answer a question:“do anaesthesia care provider show improvement on their perioperative anaesthesia information practice through simulation-based education?” based on the hypothesis that simulation-based education for perioperative anaesthesia information management has no effect on practice of anaesthesia care provider at a Comprehensive Specialized Teaching Referral Hospital in Ethiopia.

This study was conducted at a Comprehensive Specialized Teaching Referral Hospital in Ethiopia, which is far about 750 km from Northwest of Addis Ababa (the capital city of Ethiopia). This Comprehensive Specialized Teaching Referral Hospital gives surgical services in different departments. Therefore, anaesthesia care provider completed perioperative anaesthesia information records for scheduled elective and emergency cases in four operating rooms each day in the study area.

### Source population

All documented anaesthetic sheet in the study area were source population.

### Sample population

All documented anaesthetic sheet in the study area during the study period were the study population.

### Sample size determination and sampling procedure

The total number of 164 anaesthesia records of patient chart were reviewed which documented by anaesthesia care provider during study period and considered as the sample size of the study. Since we evaluated the existing practice, and service delivered by anaesthesia care provider, we assumed the sample size gives sufficient information about the manual intraoperative anaesthesia recording practice in study area.

### Operational definition

The expected completion rate was 100% for all indicators. Indicators with greater than 90% completion rate were marked as accepted, completion rate between 50 and 90% need improvement and completion rate of less than 50% was considered as areas of the critical need for improvement^4^.

**Table:** 

Indicators	Predefined components
Patients name	Name of the patients with last name
Age	Value with unit
Weight	Value with unit
Base line vital sign(V/s)	At least unit of measurment for blood presure, pulse rate, and saturation
Date of surgery	Date/month/year
Name of surgeon	Name with title and middle name
Name of Anaesthetist	Name with title and middle name
Dose/volume of administered drug	Name/dose/volume
Size of intravenous (IV) cannula	Value with unit
Site of IV cannula	Specific anatomic site
Postoperative pain management	At least assessment and management
Postoperative fluid management	Volume per h and fluid type
Total amount of blood loss	Amount with unit
Total amount of urine output	Amount and unit

### Data collection tool

Semi-structured questionnaires from the anaesthesia record format which adopted from previous study^4^, and chart review used to collect the data. To review these tools, checklist was developed based on a combination of criteria outlined by the American society of anesthesiologists (ASA),Australian and New Zealand College of Anaesthetists (ANZCA)^11^, and directly changed into question forms with three integral checking components: “Yes,” “No,” and “incomplete”.

### Data collection procedure and data analysis

A total of 328 patients’ data were collected (164 for pre-interventional and 164 for post-interventional) through chart review using structured questionnaire, which prepared for the anaesthesia record format in our set up by the principal investigators. After completion of data collection, data were coded, entered, and cleaned for errors using Epi-data software (version 4.6). Then the data has been exported and analyzed by using Statistical Package for Social Sciences (SPSS) software (version 26). Descriptive statistics were used to describe for quantitative data. The results of quantitative data were compared before and after intervention by using the frequency and percentage.

### Identified problems for the proposed actions

This study mainly identified that patient data such as documentation completeness about allergy status of the patient, about content of consent discussed, site of IV cannula, about postoperative pain management order, regarding postoperative monitoring order, about postoperative nausea and vomiting management order were Indicators critically need improvement(<50%).

### Proposed action

We carried out the following measures after analyzing the pre-interventional data, which we obtained from 164 patient charts; we arranged a half-day meeting with the department head and the clinical coordinators to discuss on identified gaps in pre-interventional phase which relate to the management of perioperative anaesthetic information and prepared a brief power point on perioperative information management from a standard perspective, identified gaps with comments from a sinour anaesthetist also we prepared leaflets about the standard. We cordially invite all anaesthesia providers taking part in the workshop.

### Implemented actions

Staff members were given short power point lecture on the basics of perioperative anaesthesia information management according to internationally accepted standards and guidelines by the principal investigator. Then we spent an hour discussion on identified gaps and disseminated the created leaflets. After distributing the standard, staff members were shown and provided constructive input on how to improve anaesthesia information documentation using the sandwich technique, allowing for a smooth facilitation of discussion. After implementing, those measure for identified gaps in pre-interventional phase for one cycle we collect data from 164 patients chart in post-interventional phase

## Results

Pre-interventional and post-interventional results on improving perioperative anaesthesia information documentation completeness. A sample of 164 manual anaesthesia records in the study area was identified. Of this sample 101 (61.5%) data’s recorded by undergraduate anaesthetist and the remain 63 (38.4%) by postgraduate and above anaesthesia service provider. This represents an overall response rate was 100%.

Among 164 manual anaesthesia records were reviewed, none of the records had 100% completion rate and induction agent, dose of induction agent, type of operation and sex of the patients had an acceptable completion rate greater than 90%.

From a total of identified indicators content of consent discussed, nothing by mouth status, age and weight of the patients were among indicators found below average (<50%) completion rate in pre-interventional phase (Table 1).

**Table 1:** Pre-intervention and post-intervention on improving anaesthetic documentation completeness about the preoperative assessment and patient’s identity

	Frequency (%)					
	Pre-intervention, *n* (%)			Post-intervention, *n* (%)		
Variables	Yes^a^	No^b^	Incomplete	Yes^a^	No^b^	Incomplete
Patients medical history	141 (86)	23 (14)	—	156 (95.1)	8 (4.9)	—
Patients physical examination	113 (68.9)	51 (31.1)	—	140 (85.4)	24 (14.6)	—
Airway examination	102 (62.2)	62 (37.8)	—	162 (98.8)	2 (1.2)	—
Allergy status of the patient	97 (59.1)	67 (40.9)	—	111 (67.7)	53 (32.3)	—
Patients history of regular medication	107 (65.2)	57 (34.8)	—	138 (84.1)	26 (15.9)	—
ASA physical status of the patient	134 (81.7)	27 (16.5)	—	148 (90.2)	16 (9.8)	—
Patient written consent taken	126 (76.8)	38 (23.2)	—	149 (90.9)	15 (9.1)	—
Content of consent discussed	15 (9.2)	145 (88.4)	—	98 (59.8)	66 (40.2)	—
NPO status of the patient	62 (37.8)	102 (66.2)	—	102 (62.2)	62 (37.8)	—
Patients name	95 (57.9)	69 (42.1)	—	115 (70.1)	9 (5.5)	—
Age of the patients	16 (9.8)	9 (5.5)	139 (84.7)	70 (42.7)	34 (20.7)	60 (36.6)
Sex of the patient	154 (93.9)	10 (6.1)	—	156 (95.1)	8 (4.9)	—
Weight	78 (47.6)	86 (52.4)	—	102 (66.2)	62 (37.8)	—
Patient chart number	141 (86)	23 (14)	—	154 (93.9)	11 (6.7)	—

ASA, American Society of Anesthesiologists; NPO, nothing by mouth.

^a^ Yes; stands for those indicators were completely practice by the participants.

^b^ No; stands for those indicators were not practice by the participants

Among indicators name of the surgeon, name of anaesthetist, site of IV cannula, maintenance of anaesthesia, pre-induction V/s, V/s throughout the procedure, total fluid administered were among indicators found below average (<50%) completion rate(Table 2).

**Table 2:** Pre-intervention and post-intervention anaesthesia information documentation completeness about operation and perioperative condition of patients

	Frequency (%)					
	Pre-interventional, *n* (%)			Post-interventional, *n* (%)		
Variables	Yes	No	Incomplete	Yes	No	Incomplete
Name of surgeon	26 (15.9)	11 (6.7)	127 (77.4)	123 (75)	30 (18.3)	11 (6.7)
Name of Anaesthetist	30 (18.3)	11 (6.7)	123 (75)	134 (81.7)	27 (16.5)	3 (1.8)
Date of operation	145 (88.4)	19 (11.6)	0	154 (93.9)	10 (6.1)	—
Type of operation	149 (90.9)	15 (9.1)	0	156 (95.1)	8 (4.9)	—
Diagnosis of operation	126 (76.8)	38 (23.2)	0	134 (81.7)	27 (16.5)	—
Anaesthetics machine and circuit checked	107 (65.2)	57 (34.8)	0	126 (76.8)	38 (23.2)	—
Size of IV cannula	91 (55.5)	73 (44.5)	0	97 (59.1)	67 (40.9)	—
Site of IV cannula	35 (21.3)	60 (36.6)	69 (42.1)	69 (42.1)	35 (21.3)	60 (36.6)
Induction agent	154 (93.9)	10 (6.1)	0	153 (93.2)	10 (6.4)	—
Dose of induction agent	153 (93.2)	10 (6.4)	1 (0.6)	115 (70.1)	9 (5.5)	40 (24.4)
Maintenance anaesthetic agent	69 (42.1)	60 (36.6)	35 (21.3)	103 (62.8)	59 (36)	2 (1.2)
Pre-induction V/s	70 (42.7)	34 (20.7)	60 (36.6)	69 (42.1)	35 (21.3)	60 (36.6)
Clear scale on v/s graph	116 (70.7)	25 (15.3)	23 (14)	123 (75)	11 (6.7)	26 (15.9)
BP,PR,SPO2,To and EtCO2	26 (15.9)	11 (6.7)	127 (77.4)	70 (42.7)	34 (20.7)	60 (36.6)
Total fluid administered	25 (15.3)	23 (14)	116 (70.7)	70 (42.7)	34 (20.7)	60 (36.6)
Total blood loss	107 (65.2)	57 (34.8)	—	111 (67.7)	53 (32.3)	—

BP, blood pressure; EtCO2, End-tidal carbon dioxide; IV, intravenous; PR, pulse rate; SpO2, saturation of peripheral oxygen; V/s, vital sign.

From a total of identified indicators site of IV cannula (42.1%), dose of induction agent (0.6%), maintenance of anaesthetic agent (21.3%), pre-induction V/s (36.6%), clear scale on V/s graph (14%), V/s throughout the procedure (77.7%), total fluid administered (70.7%), name of the surgeon (77.4%), name of anaesthetist (75%) were indicators had incomplete documentation before intervention (Table 2).

Postoperative nausea vomiting management orders were among the identified gaps for postoperative orders and management in the pre-interventional phase, and we obtained significant improvement in the post-interventional phase (1.2% vs. 65.2%) (Table 3).

**Table 3:** Pre-intervention and post-intervention anaesthesia information documentation completeness about the postoperative order and management

	Frequency (%)			
	Pre-interventional, *n* (%)		Post-interventional, *n* (%)	
Indicators	Yes	No	Yes	No
Postoperative pain management	107 (65.2)	57 (34.8)	113 (68.9)	51 (31.1)
Postoperative monitoring	95 (57.9)	69 (42.1)	98 (59.8)	66 (40.2)
PONV management order	2 (1.2)	164 (98.8)	107 (65.2)	57 (34.8)

PONV, postoperative nausea and vomiting.

## Discussion

This study showed that from all indictors none of the indicators had a completion rate of 100%. Induction agent, dose of induction agent, type of operation and sex of the patients were among indicators found acceptable (>90%) completion rate. While, postoperative nausea and vomiting management order, name of the surgeon, name of anaesthetist, site of IV cannula, maintenance of anaesthesia, pre-induction V/s, V/s throughout the procedure, total fluid administered, content of consent discussed, nothing by mouth status, age and weight of the patients were among indicators found below average (<50%) completion rate.

During pre-intervention and post-intervention weight of the patients were not recorded in 52.4% vs. 37.8% respectively. This finding was similar with previous studies from Ethiopia^2^,^9^, and Nigeria^8^, that showed a poor completion rate of demographic information of anaesthesia record tools. This indicates that the anaesthetists placed minimal emphasis on the calculation of accurate drug doses, ventilation parameters, airway equipment size, and fluid requirement, which directly affects the quality of anaesthesia delivery and perioperative patient outcome.

Pre-interventional and post-intervention pre-induction (base line vital sign) record of 42.7% and vs. 42.1% of patient was below the standard, respectively. This might affect both the intraoperative management of the patients and poor communication among different professionals. Even this study below the standard the finding of this study better compared with previous clinical audit done Ethiopia showed that pre-induction vital signs were recorded in 37.5% of patients^9^. This discrepancy might be due to improved clinical service compared with the previous one, and increasing staff members decrease staff workload.

Before intervention name of the surgeon (15.9%), name of anaesthetist (18.3%) of patients was recorded in the full name (first, second, and last name) inconsistent with previous studies^9^,^11^. While after intervention name of the surgeon (75%)and name of the anaesthetist (81.7%). The difference could have resulted from their consideration of the first and middle names as the full name of the professionals.

In this study besides, the completion rate for the age of the patient, total fluid administered, vital sign throughout the procedure and site of IV cannula were below average (<50%) even after intervention. This poor practice of documentation hugely deviated from the expected completion rate, so that, it would compromise the perioperative surgical and anaesthesia service quality, and may subsequently affect patient outcome^2^. In contrast to previous study^3^, induction agent, dose of induction agent, type of operation and sex of the patients relatively higher completion of rate were seen in our study but in line with previous audit done in Ethiopia^9^.

In present study, most of the indicators for perioperative anaesthesia information documentation were found incomplete and below the standards before intervention. Patient sociodemographic characteristics, professional’s identity, and intraoperative monitoring are among indicators found to improve completion rate after intervention but below the standard. This might be explained by workload, use of tools or manual recording, practitioner interest, and availability of information are some of the factors which affect the quality and practice of documentation^1^,^2^. In addition, anaesthesia information management system showed multiple advantages, including improved accuracy of data collection for clinical documentation and research, enhanced quality of care and improved regulatory compliance^11^,^12^. Therefore, introducing an electronic-based recording system in our case may improve patient safety, record keeping, and patient management practice.

### Strength and limitation

We have met our objectives like we identified indicators that need improvement and we aware those anaesthesia service provider about benefit of perioperative anaesthesia information management practice and we demonstrate on how they document during perioperative anaesthesia care and finally those identified indicators were improve compared with pre-interventional. However, unable to make all indicators as acceptable or 100% completion and the factors that affect information documentation not assessed was considered as limitation of the study.

### Conclusion and recommendation

In the pre-interventional phase postoperative nausea and vomiting management orders, the names of the surgeon and anaesthetist, the location of the intravenous cannula, the maintenance of anaesthesia, the total amount of fluid supplied, the content of the consent discussion, and null per ose status, age, and weight of the patient were some of the markers that were identified below average (50%) and in need of significant improvement. Even though perioperative anaesthesia information management practice was improved in post-interventional phase compared with pre-interventional phase none of the indicators attained desired completion rate. Therefore, it requires ongoing instruction on perioperative anaesthesia information management according to the standard perspectives

## Ethical approval

Ethical clearance to conduct the research was obtained from the ethical review committee of the school of Medicine and Health Sciences.

## Source of funding

Not applicable.

## Consent for publication

Not applicable.

## Authors contributions

B.M.: conceived, designed the study, supervised the data collection, performed the data analysis, interpretation of the result, and drafting the manuscript. Y.F., Z.A., M.A., and M.D.: participated in designing the study, data analysis and data interpretation, editing the manuscript. All authors read and approved the final manuscript.

## Conflicts of interest disclosure

The authors declared no conflict of interest

## Availability of data and materials

All data analyzed during this study

## Provenance and peer review

Not commissioned, externally peer-reviewed

## Acknowledgements

The authors express special gratitude to the department of anesthesia for giving them the chance to conduct this study. The authors also thank their colleagues for their constructive comments regarding the statistical issues of the study, and the whole process of the study being completed and the authors express special gratitude to study participants.
